# Edematous pink plaque in a patient on immunosuppression

**DOI:** 10.1016/j.jdcr.2023.07.008

**Published:** 2023-07-20

**Authors:** Mehvish Khan, Hunter J. Pyle, Travis W. Vandergriff, Cristina Thomas

**Affiliations:** a Department of Dermatology, The University of Texas Southwestern Medical Center, Dallas, Texas; b Department of Pathology, The University of Texas Southwestern Medical Center, Dallas, Texas; c Department of Internal Medicine, The University of Texas Southwestern Medical Center, Dallas, Texas

**Keywords:** atypical cellulitis, cellulitis, cutaneous histoplasmosis, disseminated histoplasmosis, infectious disease, medical dermatology

A 61-year-old male in Texas presented with 13 months of painful, erythematous swelling of his left arm associated with cough, lymphadenopathy, and migratory polyarthralgias. Cough workup 6 months prior demonstrated calcified lung nodules on chest computed tomography and noncaseating granulomas on lung biopsy. He received 5 months of azathioprine and prednisone for presumed sarcoidosis without improvement. Examination demonstrated an indurated erythematous plaque from the left forearm to dorsal hand on a background of edema (Fig 1). Punch biopsy was obtained due to persistence of the lesion despite broad-spectrum antibiotics. Grocott methenamine silver stain demonstrated dermal narrow-based budding yeast (Fig 2).

**Fig 1:**
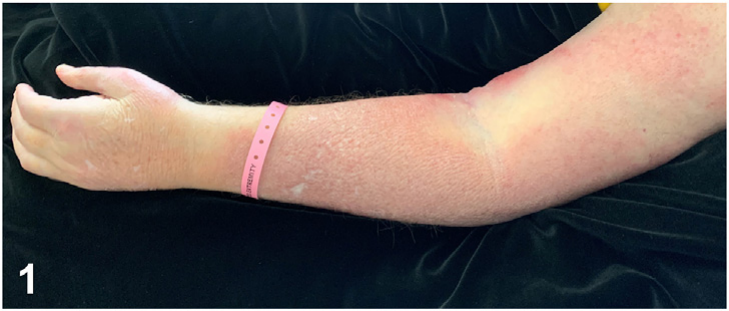

**Fig 2:**
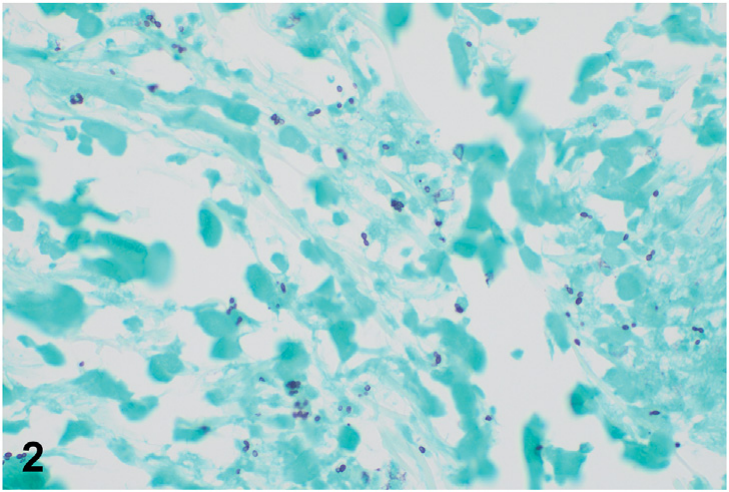

**Question 1: What is the most likely cause for his cutaneous findings?**

A. Bacterial cellulitis
B. Sarcoidosis
C. Disseminated histoplasmosis
D. Coccidioidomycosis
E. Aspergillosis

**Answers:**

A. Bacterial cellulitis – Incorrect. Grocott methenamine staining (Fig 2) of the skin biopsy highlighted small narrow-based budding yeast in the dermis consistent with histoplasmosis. Bacterial cellulitis would not show yeast on pathology.
B. Sarcoidosis – Incorrect. Although sarcoidosis can present with subcutaneous nodules as well as systemic symptoms such as fevers, the patient’s resistance to immunosuppression and presence of narrow-based budding on skin biopsy (Fig 2) indicates another cause.
C. Disseminated histoplasmosis – Correct. Lymphadenopathy and pulmonary findings unresponsive to immunosuppression suggest infection. In disseminated histoplasmosis, pulmonary involvement, lymphadenopathy, and hepatosplenomegaly are common. Cutaneous manifestations of disseminated disease are less common, and lesions can be polymorphous, including umbilicated papules, nodules, ulcers, pustules, vesicles, and erythroderma. Much more rarely, disseminated disease has been reported to present with cellulitis-like changes in patients on chronic immunosuppressive therapy.^1^ Histoplasmosis pseudocellulitis should be considered in patients living in or with recent travel to endemic areas with persistent skin findings despite antibiotics.
D. Coccidioidomycosis – Incorrect. *Coccidioides* infection predominantly starts in the lungs. Dissemination to the skin, similar to histoplasmosis, can be seen in immunocompromised patients. Pathology would demonstrate spherules containing endospores within granulomas.^2^
E. Aspergillosis – Incorrect. *Aspergillus* is a widely prevalent organism present in soil and stored grains. The disseminated form is most common in immunocompromised patients. However, skin biopsy findings would show septate hyphae branched at a 45-degree angle.^3^

**Question 2: What is the most common risk factor for the development of this diagnosis?**

A. Living in the southwestern United States
B. Occupational history of working as a sandblaster
C. Atopic dermatitis
D. Family history
E. Immunosuppression

**Answers:**

A. Living in the southwestern United States – Incorrect. Histoplasmosis is the most prevalent of the major endemic mycoses in the United States, with cases most commonly reported in the Ohio and Mississippi River Valleys but also occurring in Texas among other regions.^4^ Coccidioidomycosis is more common in the southwestern United States.
B. Occupational history of working as a sandblaster – Incorrect. Silicosis can be associated with pulmonary disease in sandblasters as a result of inhalation of silica. Systemic sclerosis can be seen with silicosis, but cellulitis-like changes have not been reported.
C. Atopic dermatitis – Incorrect. Atopic dermatitis can lead to skin fissuring that allows for bacterial translocation and cellulitis. However, atopic dermatitis is not a risk factor for histoplasmosis.
D. Family history – Incorrect. Although a family history of sarcoidosis is a risk factor for sarcoidosis, disseminated histoplasmosis is not thought to be due to genetic factors.
E. Immunosuppression – Correct. This patient’s history of iatrogenic immunosuppression has most likely led to disseminated histoplasmosis with cutaneous findings. CD4^+^ and CD8^+^ T cells are required for an effective immune response in histoplasmosis. As such, disseminated histoplasmosis is more common in those with T-cell immunodeficiencies, iatrogenic T-cell suppression, or acquired T-cell deficiencies such as HIV.^5^

**Question 3: What is the most appropriate treatment for this patient?**

A. Intravenous corticosteroids
B. Intravenous amphotericin B and/or an azole
C. Intravenous antibiotics
D. Incision and drainage
E. Infliximab

**Answers:**

A. Intravenous corticosteroids – Incorrect. Presumably, this patient’s initial fevers, pulmonary nodules, and arthralgias were due to undiagnosed histoplasmosis rather than sarcoidosis and worsening of his symptoms occurred as a result of immunosuppressive therapy. Increasing immunosuppression would not result in improvement.
B. Intravenous amphotericin B and/or an azole – Correct. Because mortality can be as high as 80% to 100% in disseminated histoplasmosis, all patients should be treated with intravenous amphotericin B and/or an azole.^5^ The specific choice of drug depends on disease severity and the presence of central nervous system involvement, but in all cases, treatment should be continued for at least 12 m.
C. Intravenous antibiotics – Incorrect. Bacterial cellulitis is not suspected given the skin biopsy findings, and thus, intravenous antibiotics are not warranted.
D. Incision and drainage – Incorrect. Incision and drainage would be warranted if there was concern for an abscess. However, this would not improve disseminated histoplasmosis, and instead, antifungal therapy is required.
E. Infliximab – Incorrect. Infliximab and other tumor necrosis factor-alpha inhibitors can be used in the treatment of sarcoidosis. However, further immunosuppression in this patient would worsen disseminated histoplasmosis.

## Conflicts of interest

None disclosed.
